# Generative adversarial networks-based Image-to-Image translation allows tumor consistency prediction from standard MR images in pituitary adenomas

**DOI:** 10.1371/journal.pdig.0001407

**Published:** 2026-05-13

**Authors:** Guoqing Wu, Shun Yao, Xiaohai Liu, Wenting Rui, Hanning Xu, Zengyi Ma, Yongfei Wang, Zhenwei Yao, Ge Chen, Ying Guo, Haiyong He, Nidan Qiao, Jinhua Yu, Yao Zhao

**Affiliations:** 1 AI Lab of Department of Neurosurgery, Huashan Hospital, Shanghai Medical College, Fudan University, Shanghai, China; 2 School of Information Science and Technology, Fudan University, Shanghai, China; 3 Department of Neurosurgery, Huashan Hospital, Shanghai Medical College, Fudan University, Shanghai, China; 4 National Center for Neurological Disorders, Shanghai, China; 5 Shanghai Key laboratory of Brain Function Restoration and Neural Regeneration, Shanghai, China; 6 Neurosurgical Institute of Fudan University, Shanghai, China; 7 Department of Neurosurgery, Xuanwu Hospital, Capital Medical University, Beijing, China; 8 Department of Radiology, Huashan Hospital, Shanghai Medical College, Fudan University, Shanghai, China; 9 The Third Affiliated Hospital, Sun Yat-Sen University, Guangzhou, China; Tianjin Medical University General Hospital, CHINA

## Abstract

Generative adversarial networks (GANs) offer potential in cross-modality image translation, but their application in pituitary adenomas remains uncertain. This study was to assess the feasibility of a GAN-based deep learning algorithm for generating synthetic diffusion-weighted imaging (DWI) associated images and its clinical utility in predicting tumor consistency. This multicenter study included a training cohort of 152 participants with large-to-giant pituitary adenomas from a tertiary center. Synthetic DWI associated images were generated from T2-weighted images (T2WI) and evaluated against real images. External validation was performed on a cohort of 69 participants from three additional centers to assess the utility of synthetic images in predicting tumor consistency and their correlation with surgical outcomes. In the independent test set, synthetic images demonstrated close resemblance to real images, with a mean squared error (MSE) below 70 and peak signal-to-noise ratio (PSNR) exceeding 30 dB. Neuroradiologists were largely unable to distinguish between real and synthetic images (P = 0.571) and rated comparable scores in the overall image quality (P = 0.051) and diagnostic confidence (P = 0.168) scale. Synthetic images demonstrate comparable performance to real images in predicting tumor consistency in both the independent test set (AUC = 0.84) and external cohort (AUC = 0.79). When combining features from all the three synthetic images, the performance in predicting tumor consistency outperformed conventional T2WI (P = 0.036 in the test set, P = 0.040 in the external cohort). Surgeons were more likely to adopt transcranial or combined approaches for predicted fibrous tumors, which were associated with lower tumor removal rates and higher severe complication rates compared to soft tumors. The GAN-based image synthesis method accurately predicts tumor consistency and shows potential for guiding surgical decision-making in patients with pituitary adenomas.

## Author summary

In this study, we explored whether a type of artificial intelligence (AI) could create realistic medical scans to help surgeons better plan operations for pituitary tumors. Specifically, we used this AI to generate special MRI scans that are typically hard to get, from a more common type of MRI. We tested this approach on patients with pituitary adenoma from multiple hospitals. Our results show that the AI-generated images were remarkably similar to real ones and were just as effective at predicting whether a tumor is soft or firm—a key factor in surgical planning. Importantly, when surgeons had this prediction, they chose more appropriate surgical strategies. This work demonstrates that AI-synthesized images can be a valuable and reliable tool, potentially helping neurosurgeons make better decisions and improve patient outcomes.

## Introduction

Pituitary adenomas (PAs) are among the most common lesions of the sella turcica, comprising 10% to 25% of all intracranial neoplasms [1]. Surgical intervention remains the primary treatment modality for PAs, with the exception of prolactinomas, which are typically managed with medical therapy. The consistency of the tumor is a critical factor influencing the complexity of surgical resection [2,3]. Tumors characterized by a tenacious consistency pose significant challenges during surgery, necessitating the use of specialized instruments, such as scissors, to facilitate removal. This increased difficulty can result in the heightened risk of complications and incomplete tumor resection, ultimately impacting patient outcomes [4].

Accurately predicting the difficulty of pituitary adenoma surgery through preoperative imaging techniques is vital for developing a comprehensive surgical plan. Such predictions can inform modifications to the surgical approach, maximizing tumor resection while prioritizing patient safety. For patients, an objective evaluation of the surgery’s complexity, potential outcomes, and associated risks prior to the procedure is crucial in guiding their choice of the most suitable treatment center. Therefore, there is an urgent need for reliable preoperative assessments of tumor consistency.

Magnetic Resonance (MR) imaging is an essential tool for diagnosing pituitary adenomas, with diffusion-weighted imaging (DWI) modality recognized as a well-established technique for evaluating tumor consistency [5–9]. Despite its clinical value, this technique faces several limitations, including the requirement for specialized imaging protocols, prolonged acquisition times, and the complexity of its application.

Image-to-image translation, which transforms one visual style into another or creates a mapping between different image types, presents a potential solution by converting standard MR scans into more advanced imaging representations. Generative adversarial networks (GANs), a deep learning-based framework, learn mappings from training data and generate synthetic data that closely mirrors the characteristics of the original dataset. GANs and their variants have been extensively applied in brain image synthesis, demonstrating significant promise in cross-modality image translation [10–12]. This innovative approach not only enhances the quality of preoperative assessments but also holds the potential to improve clinical decision-making in the management of tumors.

Our study aimed to evaluate the feasibility of a GAN-based deep learning algorithm for generating images in the DWI modality from standard MR scans. In addition, we sought to assess the clinical utility of these synthetic images in predicting tumor consistency, surgical difficulty, and prognosis. To ensure robustness and generalizability across diverse clinical settings, the evaluation was conducted using multicenter external cohort.

## Materials and methods

### Study sample

**Fig 1:**
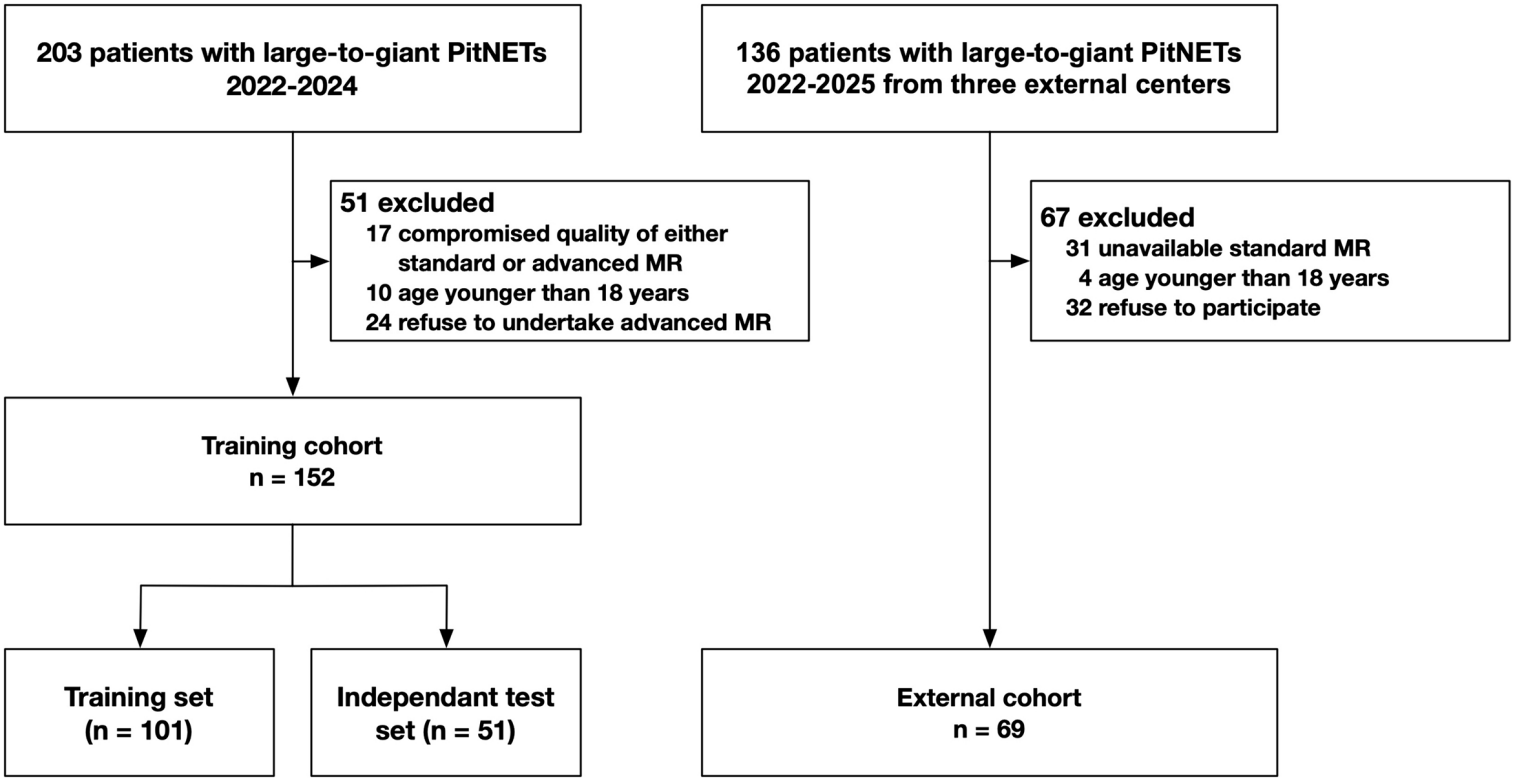
Flowchart shows inclusion of study participants in both the training cohort and external cohort.

**Fig 2:**
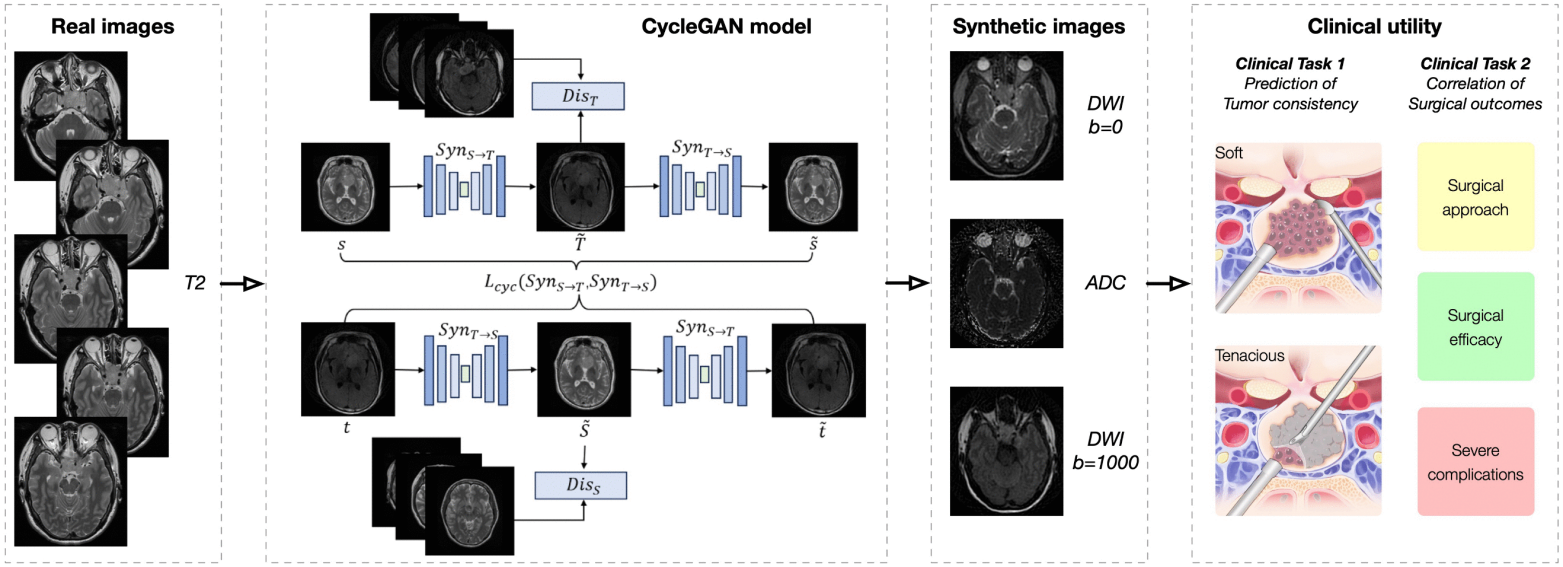
Real T2-weighted images (T2WI) were processed using an end-to-end CycleGAN-based model to generate synthetic diffusion-weighted imaging (DWI)with b = 0 and b = 1000, and apparent diffusion coefficient (ADC) images. These synthetic images were then employed to predict tumor consistency and analyze correlations with surgical approaches, outcomes, and complications.

In this observational study, consecutive patients with large-to-giant pituitary adenomas were screened for inclusion between December 2022 and May 2024 (Fig 1). Exclusion criteria included (a) compromised quality of either standard or advanced MR examinations and (b) age younger than 18 years. The external cohort were retrospectively collected, consisting 69 patients treated at three external centers. All experiments were conducted in accordance with the Declaration of Helsinki. The study was approved by our Institutional Review Board (KY2022–709), and informed consent was obtained from all participants. The study was registered on Clinicaltrials.gov (NCT06664190). The study framework is presented in Fig 2.

### MR image acquisition

MR images were acquired using 3.0 Tesla scanners (Discovery MR 750W; GE Medical Systems) equipped with 24-channel head matrix coils. Each patient underwent multi-parametric MR scanning prior to surgery, including conventional sequences (axial T1-weighted imaging [T1WI], axial T2-weighted imaging [T2WI], and contrast-enhanced axial T1WI) as well as advanced sequences (DWI). The parameters for the MR sequences were as follows:

- T1WI: repetition time/ echo time 400/12 ms, field of view 22 cm, matrix size 256*224, bandwidth 62.5 kHz, echo train length 30, and slice thickness 2 mm;
- T2WI: repetition time/ echo time 2500/60 ms, field of view 22 cm, matrix size 320*320, bandwidth 62.5 kHz, echo train length 60, and slice thickness 2 mm;
- Contrast-enhanced axial T1WI was performed immediately after administering a standard dose (0.1 mmol/kg) of gadopentetate dimeglumine (Beilu) at approximately 3–4 mL/s via the dorsal hand or elbow vein.
- DWI was acquired with b-value of 0: 1000 sec/mm2, repetition time/ echo time 5438/85 ms, field of view 21 cm, bandwidth 83.3 kHz, echo train length 16, NEX 1.5, and slice thickness 3 mm; the raw data were transferred to a workstation (Advantage workstation 4.6, GE Medical Systems) using the Functool software (GE Medical Systems) to calculate and generate images with a b-value of 1000 and apparent diffusion coefficient (ADC) images.

### Image-to-Image translation model

GANs typically consist of two components: a generator and a discriminator, both of which are neural networks that engage in a competitive learning process. The generator receives source data and is trained to produce synthetic (fake) target data that resembles real target data. Simultaneously, the discriminator is trained to differentiate between the real target data and the synthetic fake data. Through this adversarial process, the generator improves its ability to create increasingly realistic synthetic data, while the discriminator becomes more proficient at distinguishing between real and generated data.

An end-to-end CycleGAN-based model [13] is introduced as a basic framework to synthesize virtual MR images by facilitating mutual conversion between two imaging modalities. CycleGAN establishes two cycles of transformations to achieve this. In the first cycle, the original modality is converted to the target modality and back to the original, handled by two generator networks, Syn_S→T_ and Syn_T→S_, respectively. In the second cycle, the target modality is converted back to the original modality and then returned to the target, using the same generator networks Syn_T→S_ and Syn_S→T_. Importantly, the same generators are employed for both cycles, maintaining consistency in the conversion process. Additionally, two discriminator networks, Dis_T_ and Dis_S_, are trained to differentiate between the synthetic modality and the real modality. During the training phase, the interaction between the generator and discriminator networks through adversarial training enhances the quality of the synthesized images, making them more accurate and realistic. We used an Intel(R) Xeon(R) Gold 6128 CPU @ 3.40 GHz and an NVIDIA A10 GPU (24 GB VRAM) to train the model.

In this study, the original modality is T2WI, and the target modalities include DWI (b = 0 and b = 1000) and ADC images. To improve the quality of the synthetic images and bring them closer to real images, the model employs an enhanced U-Net architecture with attention residual blocks as the core component of the generator. S1 Method provides detailed information on the network structures of both the generator and discriminator, the total loss function employed, model training parameters, as well as the software and hardware environment used for model implementation (S1 Fig and S2 Fig).

### Evaluation of synthetic images

For quantitative evaluation, we employed Mean Squared Error (MSE) and Peak Signal-to-Noise Ratio (PSNR) metrics to assess the quality of the synthetic images. MSE measures the pixel-wise similarity between the synthesized and original MRI images, with lower values indicating higher fidelity (an ideal reconstruction has an MSE < 80). PSNR complements this measure, with values greater than 30 dB suggesting high-quality reconstruction.

For qualitative evaluation, 50 randomly selected images (25 real and 25 synthetic) from each modality (in the test set) were assigned to two neuroradiologists (W.R. and Z.Y., with 10 and 12 years of MR experience, respectively). Both radiologists were blinded to the nature of the images and independently tasked with determining whether each presented image was real or synthetic. Furthermore, the two neuroradiologists rated both the real and synthetic images using the following scales. The overall image quality was rated on a scale from 1 to 4, with 1 indicating poor quality, 2 indicating moderate quality, 3 indicating good quality, and 4 indicating excellent quality. Diagnostic confidence was assessed using a four-point scale: a score of 1 indicated inadequate assessment of any pathologies; a score of 2 indicated possible lesion detection with moderate suspicion; a score of 3 indicated good lesion detection with high suspicion; and a score of 4 indicated excellent lesion detection with very high suspicion [14].

### The value of synthetic images in clinical practice

We assessed the utility of synthetic images in predicting tumor consistency. In each participating center, the lead surgeon intraoperatively assessed and recorded tumor texture in the surgical notes, using the classification method established in the literature [15]. Tumors were graded as either soft (primarily amenable to suction removal) or fibrous (resistant to suction and requiring sharp dissection with scissors or other mechanical techniques).

Then, tumors were labeled by two researchers (S.Y. and W.R.) using the itk-snap tool with reference to contrast-enhanced T1WI. Inter-rater reliability for these segmentations was excellent, as quantified by high Dice similarity coefficients (T2-weighted images: 0.951; ADC maps: 0.881). Subsequently, a total of 526 features were extracted from the tumor regions of each MR modality (T2WI, DWI b = 0, DWI b = 1000, and ADC), categorized into four types: 13 shape features, 18 intensity features, 39 texture features, and 456 wavelet features. This feature extraction was conducted using a MATLAB-based radiomics feature extraction package [16].

To prevent potential information leakage during feature selection, the dataset was first divided into training and independent test sets in a 2:1 ratio (101 as training set and 51 as independent test set). All feature selection procedures were conducted exclusively on the training data, and the independent test set was not accessed until the final model evaluation stage. A two-stage feature selection strategy was adopted based solely on the training set. First, a sparse representation–based feature ranking method [17] was applied to rank features according to their discriminative importance. Subsequently, a sequential forward selection procedure was used to determine the optimal feature subset. During this forward-selection process, an inner sparse-representation classifier was embedded to evaluate candidate feature subsets using 10-fold cross-validation on the training set. The cross-validated classification performance served as the criterion for selecting the final feature subset. No information from the independent test set was used at any stage of feature ranking or selection. After the optimal feature subset (S3 Fig) was identified, a multilayer perceptron classifier was trained on the training set using only the selected features and then evaluated on the independent test set.

The performance of the model was evaluated using several metrics, including accuracy, sensitivity, specificity, receiver operating characteristic (ROC) curve, and the area under the ROC curve (AUC). These metrics were applied to assess the model’s performance in the independent test set as well as in cases from external centers.

The predicted tumor consistency was further correlated with surgical approaches, outcomes and complications. Gross Total Resection (GTR) was defined as the absence of residual tumor on postoperative contrast-enhanced T1-weighted imaging. Severe postoperative complications included intracranial hemorrhage, major vascular injury, ischemic stroke, cranial nerve palsy, intracranial infection, postoperative hydrocephalus, cerebrospinal fluid leakage, unplanned reoperation, decreased consciousness score, or death.

### Statistical analysis

Quality between the real and synthetic images was compared using Wilcoxon signed rank test. The AUC for each MRI modality was computed and compared (e.g., synthetic DWI versus original T2WI) for statistical significance. Additionally, we conducted category-free net reclassification index (NRI) and integrated discrimination improvement (IDI) analyses to provide further validation of our findings. Differences between AUCs were assessed using DeLong’s test. A two-sided P-value of <.05 was considered statistically significant. Statistical analyses were performed by N.Q., who has a statistical degree and six years of experience, using R software (version 3.2.0, www.r-project.org).

## Results

### Study sample

A tot‌‌al of 152 patients (88 male) were divided into a training set (n = 101) and an independent test set (n = 51). Nearly half were diagnosed with giant pituitary adenomas, providing sufficient imaging area for model training. Among the participants, 64 were classified as having fibrous tumors, while 88 had soft tumors. Additionally, 69 patients from three external centers were included in the external validation cohort. The study flowchart is presented in Fig 1, and detailed demographic and clinical information is summarized in Table 1.

**Table 1:** Clinical characteristics of the study cohort.

	Training cohort (N = 152)	External cohort (N = 69)
**Sex**		
Male	88 (57.9%)	31 (44.9%)
Female	64 (42.1%)	38 (55.1%)
**Age (years old)**		
< 35	34 (22.4%)	10 (14.5%)
>= 35 & < 60	85 (55.9%)	40 (58.0%)
>= 60	33 (21.7%)	19 (27.5%)
**Tumor consistency**		
Fibrous	64 (42.1%)	18 (26.1%)
Soft	88 (57.9%)	51 (73.9%)
**Pituitary surgical history**		
No	124 (81.6%)	63 (91.3%)
Yes	28 (18.4%)	6 (8.7%)
**Tumor volume**		
Large	77 (50.7%)	55 (79.7%)
Giant	75 (49.3%)	14 (20.3%)
**Clinical subtype**		
Non-functional	115 (75.7%)	54 (78.3%)
Functional	37 (24.3%)	15 (21.7%)
**Tumor invasion**		
Cavernous sinus (Knosp 3–4)	98 (64.5%)	29 (42.0%)
Suprasellar	58 (38.2%)	19 (27.5%)
Sphenoid sinus/clivus	57 (37.5%)	17 (24.6%)
Willis Circle	23 (15.1%)	11 (15.9%)

### Quantitative evaluation of the synthesized MRI

**Fig 3:**
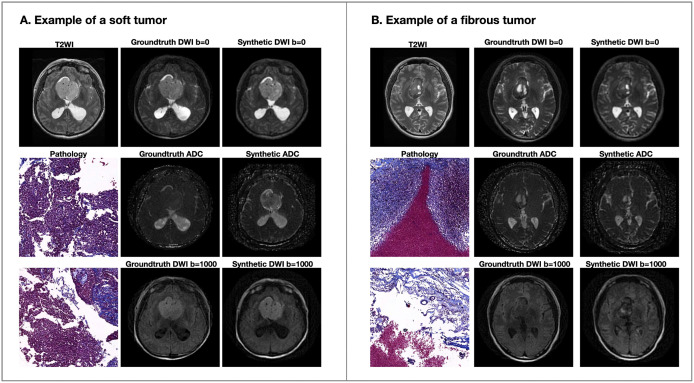
T2WI, pathology images, ground-truth, and synthetic images in patients with pituitary adenomas. **(A)** Example of a soft tumor: Masson’s trichrome staining revealed abundant tumor tissue with minimal fibrous tissue (blue color). **(B)** Example of a fibrous tumor: Masson’s trichrome staining revealed abundant fibrous tissue (blue color). Magnification: × 100. DWI = diffusion-weighted imaging; ADC = apparent diffusion coefficient; T2WI = T2-weighted image. Magnetic resonance images are displayed after the grayscale values are uniformly normalized to the range of 0-255 based on the window width and window level settings.

The synthetic images generated by this model closely resembled real images in appearance. Fig 3A and 3B illustrate examples of a fibrous and a soft tumor, where the fibrous tumor demonstrated a higher density of fibrous tissue, as confirmed by Masson’s trichrome staining.

Table 2 summarizes the quantitative results for the synthetic images in the independent test set. The MSE during the conversion of T2WI images to DWI b = 0, ADC or DWI b = 1000 was consistently under 70, indicating accurate translation across modalities. Additionally, the PSNR values exceeded 30 dB in both DWI b = 0 and ADC images, further reflecting the model’s strong performance in producing high-fidelity synthetic images.

**Table 2:** Similarity results of the synthetic images.

	MSE [95% CI]	PSNR [95% CI]	SSIM [95% CI]
Synthetic DWI b = 0	56.3 [44.9-67.7]	30.6 [29.8-31.5]	93.4 [90.7.-96.1]
Synthetic ADC	31.4 [22.7-40.1]	33.2 [32.0-34.4]	95.8 [93.6-98.0]
Synthetic DWI b = 1000	72.5 [64.6-80.5]	29.5 [29.1-30.0]	92.6 [89.3-95.9]

MSE: Mean Squared Error; PSNR: Peak Signal-to-Noise Ratio; SSIM: structural similarity. MSE < 80 indicates an ideal reconstruction, PSNR > 30 dB suggests high-quality reconstruction and SSIM > 90 indicates good structural consistency between the reconstructed image and the ground truth image.

### Qualitative evaluation of the synthesized MRI

In the blinding test, we randomly selected 50 images (25 real and 25 synthetic) from each modality (in the independent test set) to evaluate the ability of two experienced neuroradiologists to differentiate between them. The results demonstrated that the radiologists showed a strong tendency to label images as real. Inter-reader agreement (intraclass correlation coefficient, ICC) for determining synthetic images was 0.85 (95% CI: 0.73–0.92). Among the real images, 16.7% (IQR: 14.0%–19.0%) were incorrectly identified as synthetic. Similarly, among the synthetic images, only 19.3% (IQR: 16.3%–20.0%) were correctly identified as synthetic (P = 0.571, Table 3). The neuroradiologists’ performance in classifying synthetic images was as follows: Neuroradiologist 1 achieved an accuracy of 0.487, a sensitivity of 0.173, and a specificity of 0.800; Neuroradiologist 2 achieved an accuracy of 0.520, a sensitivity of 0.200, and a specificity of 0.840.

**Table 3:** Confusion matrix of the “real or fake” test by two radiologists.

		Ground truth					
		DWI b = 0		ADC		DWI b = 1000	
		Real	Synthetic	Real	Synthetic	Real	Synthetic
**Rating of Radiologist 1**	Real	21	20	18	21	21	20
	Synthetic	4	5	7	4	4	5
**Rating of Radiologist 2**	Real	22	20	21	19	21	21
	Synthetic	3	5	4	6	4	4

**Fig 4:**
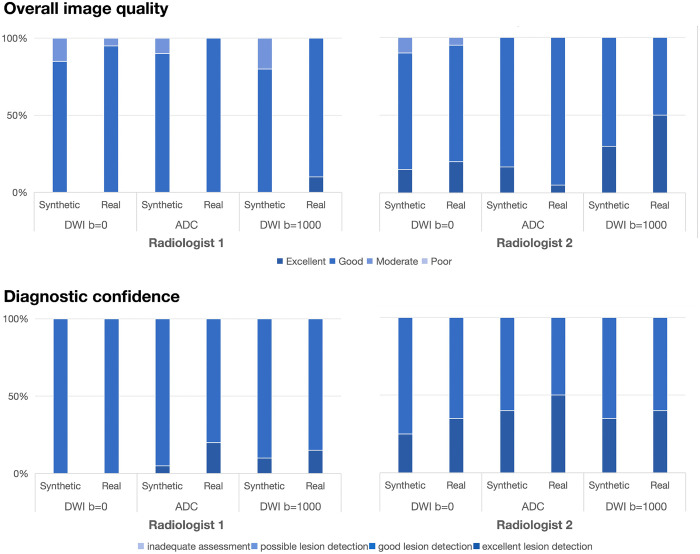
Distributions of the rating scale by two neuroradiologists for overall image quality and diagnostic confidence.

Using rating scales, both radiologists assigned scores of 3 or 4 for overall image quality to nearly all synthetic images. The overall image quality of synthetic images was noninferior to that of real images (Fig 4; pooled mean rating, 2.96 ± 0.71 vs. 3.12 ± 0.44; P = 0.051). Similarly, the diagnostic confidence scores for synthetic images were comparable to those of real images (pooled mean rating, 3.19 ± 0.43 vs. 3.26 ± 0.67; P = 0.168), indicating that the synthetic images provided equivalent diagnostic utility.

**Fig 5:**
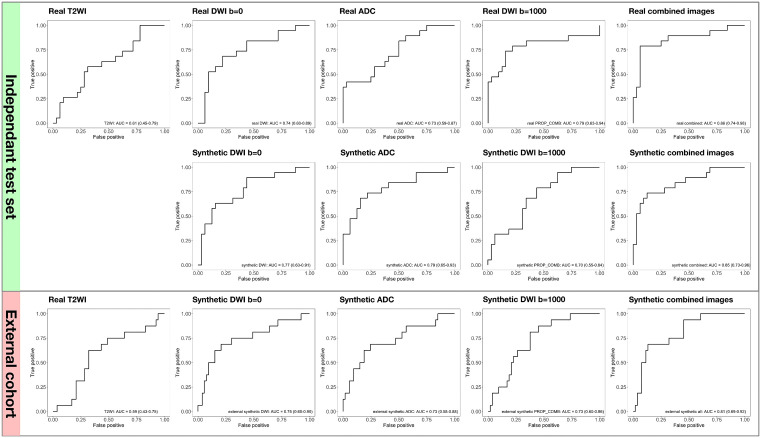
Receiver operating characteristic (ROC) curves and corresponding area under the curve (AUC) values for each imaging modality, including both real and synthetic images, in predicting tumor consistency.

The average end-to-end processing time for per case, including data loading and T2W-to-DWI, was 0.25 seconds, significantly faster than the average duration of over one minute required‌‌ for real scans.

**Fig 6:**
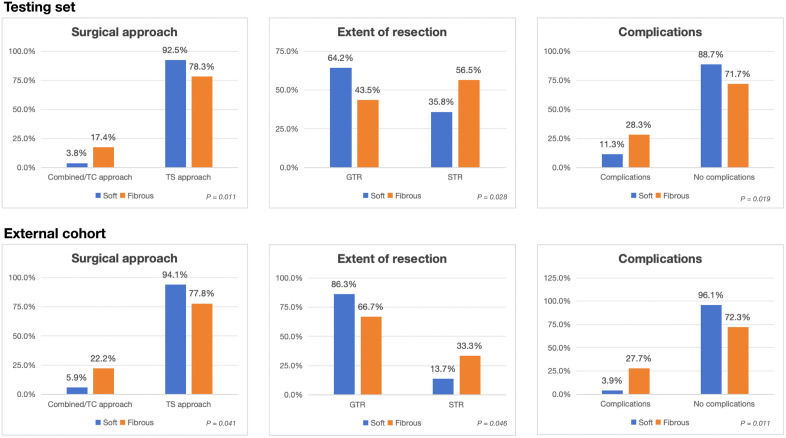
Correlation of tumor consistency with surgical approaches, outcomes and risks. TC = transcranial; TS = transsphenoidal.

### Clinical values of synthetic images

In the independent test set, the AUC for predicting tumor consistency using T2WI was 0.61 (95% CI: 0.45–0.79). The AUC for tumor consistency prediction with synthetic DWI b = 0, ADC, and DWI b = 1000 was 0.77, 0.79, and 0.70, respectively. When we combined features from all three synthetic images, the prediction performance increased to 0.84. These values were comparable to those obtained with real DWI associated images. Notably, the use of synthetic images significantly improved predictive accuracy compared to conventional T2WI (P = 0.039; Table 4). Prediction using the combined features from all the synthetic images demonstrated an NRI of 0.61 (95% CI: 0.29–0.92, P < 0.01) compared to real T2WI (Fig 5). The corresponding IDI was 0.33 (95% CI: 0.17–0.48, P < 0.01). The selected features and their importance rankings are presented in S3 Fig.

**Table 4:** Clinical utility of the synthetic images in predicting tumor consistency.

Independent test set					
	T2WI	Real DWI b = 0	Real ADC	Real DWI b = 1000	Real Combined
AUC	0.613 [0.452 - 0.775]	0.743 [0.597 - 0.890]	0.730 [0.586 - 0.875]	0.789 [0.634 - 0.945]	0.860 [0.741 - 0.980]
Accuracy	0.647 [0.630 - 0.664]	0.745 [0.725 - 0.765]	0.765 [0.743 - 0.783]	0.804 [0.784 - 0.824]	0.882 [0.861 - 0.903]
Sensitivity	0.579 [0.357 - 0.801]	0.684 [0.475 - 0.893]	0.421 [0.199 - 0.643]	0.737 [0.539 - 0.935]	0.789 [0.606 - 0.973]
Specificity	0.688 [0.527 - 0.848]	0.781 [0.638 - 0.924]	0.969 [0.908 - 1.000]	0.844 [0.718 - 0.970]	0.938 [0.854 - 1.000]
		**Synthetic DWI b = 0**	**Synthetic ADC**	**Synthetic DWI b = 1000**	**Synthetic Combined**
AUC		0.768 [0.630 - 0.907]	0.791 [0.653 - 0.929]	0.696 [0.550 - 0.842]	0.842 [0.725 - 0.959]
Accuracy		0.765 [0.748 - 0.782]	0.784 [0.770 - 0.798]	0.647 [0.630 - 0.664]	0.824 [0.800 - 0.848]
Sensitivity		0.632 [0.415 - 0.848]	0.684 [0.475 - 0.893]	0.789 [0.606 - 0.973]	0.737 [0.539 - 0.935]
Specificity		0.844 [0.718 - 0.970]	0.844 [0.718 - 0.970]	0.562 [0.391 - 0.734]	0.875 [0.760 - 0.990]
	**Synthetic Combined vs Real T2WI**	**Real vs Synthetic DWI b = 0**	**Real vs Synthetic ADC**	**Real vs Synthetic** **DWI b = 1000**	**Real vs Synthetic Combined**
P value	0.039	0.812	0.550	0.385	0.831
**External cohort**					
	**T2WI**	**Synthetic DWI b = 0**	**Synthetic ADC**	**Synthetic DWI b = 1000**	**Synthetic Combined**
AUC	0.586 [0.425 - 0.747]	0.748 [0.596 - 0.899]	0.729 [0.576 - 0.882]	0.729 [0.601 - 0.857]	0.805 [0.690 - 0.921]
Accuracy	0.667 [0.650 - 0.684]	0.768 [0.748 - 0.788]	0.739 [0.720 - 0.758]	0.667 [0.651 - 0.683]	0.826 [0.800 - 0.852]
Sensitivity	0.625 [0.388 – 0.862]	0.688 [0.460 - 0.915]	0.688 [0.460 - 0.915]	0.812 [0.621 - 1.000]	0.688 [0.460 - 0.915]
Specificity	0.679 [0.554 - 0.805]	0.792 [0.683 - 0.902]	0.755 [0.639 - 0.871]	0.623 [0.492 - 0.753]	0.868 [0.777 - 0.959]
		**Synthetic DWI b = 0 vs T2WI**	**Synthetic ADC** **vs T2WI**	**Synthetic DWI b = 1000** **vs T2WI**	**Synthetic Combined vs T2WI**
P value		0.159	0.151	0.218	0.040

In the external cohort, the accuracy for predicting tumor consistency using synthetic DWI b = 0, ADC, and DWI b = 1000 was 0.75, 0.73, and 0.73, respectively. Combined features from all three synthetic images obtained an AUC of 0.81. This result was significantly higher than the accuracy achieved with real T2WI alone (0.59, 95% CI: 0.43–0.75, P = 0.040; Table 4). The NRI using the combined features from all the synthetic images was 0.33 (95%CI: -0.06 - 0.72, P = 0.101) comparing to real T2WI, and the corresponding IDI was 0.21 (95%CI: 0.07 - 0.35, P < 0.01). Calibration curves (S4 Fig and S5 Fig) and decision curves (S6 Fig and S7 Fig) were presents in Supplements.

Regarding the prediction of surgical outcomes, analysis in the training cohort revealed that surgeons were more likely to adopt a transcranial or combined approach for patients with predicted fibrous tumors compared to a conventional transsphenoidal approach (P = 0.010). Patients with predicted fibrous tumors demonstrated a lower rate of tumor removal (43.5%) compared to those with soft tumors (64.2%, P = 0.028). Additionally, patients with fibrous tumors experienced a higher rate of severe complications (28.3%, P = 0.019). Similar outcome disparities were observed in the external cohorts, further validating these findings (Fig 6). Multivariable analysis, adjusted for surgical history, tumor size, cavernous sinus invasion, and suprasellar invasion, indicates that tumor consistency was an independent risk factor for these outcomes (Table 5).

**Table 5:** Multivariable analysis.

Independent test set		Outcome: Approach Combined/Transcranial vs. TS		Outcome: resection extent Gross total vs. Subtotal		Outcome: complication Yes vs. No	
		OR	p	OR	p	OR	p
Consistency	Soft	Reference	0.004*	Reference	0.012*	Reference	0.004*
	Fibrous	10.2 [2.34 -57.7]		0.35 [0.15 - 0.78]		4.76 [1.70 – 14.2]	
Surgical history	No	Reference	0.195	Reference	0.010*	Reference	0.187
	Yes	0.20 [0.09-1.58]		0.26 [0.09 - 0.70]		2.21 [0.66 – 7.12]	
Tumor diameter	< 4 cm	Reference	0.705	Reference	0.095	Reference	0.035*
	≥4 cm	1.46 [0.21 -12.8]		0.48 [0.20 - 1.12]		3.82 [1.16 – 14.5]	
Cavernous sinus invasion	No	Reference	0.221	Reference	<0.001*	Reference	0.584
	Yes	3.28 [0.58 – 29.8]		0.13 [0.05 - 0.31]		1.39 [0.45 -4.79]	
Suprasellar invasion	No	Reference	0.002*	Reference	0.247	Reference	0.003*
	Yes	6.19 [2.20 – 22.7]		1.38 [0.80 - 2.45]		2.66 [1.40 – 5.25]	
**External cohort**							
Consistency	Soft	Reference	0.028*	Reference	0.039*	Reference	0.013*
	Fibrous	30.7 [2.39 - 150]		0.16 [0.02 – 0.86]		31.6 [3.26 - 120]	
Surgical history	No	Reference	0.612	Reference	0.113	Reference	0.750
	Yes	2.16 [0.97 – 5.08]		0.08 [0.01 – 1.85]		0.59 [0.01 – 12.9]	
Tumor diameter	< 4 cm	Reference	0.491	Reference	0.994	Reference	0.488
	≥4 cm	2.42 [2.07 – 3.86]		0.11 [0.02 - 10.1]		0.37 [0.01 – 5.83]	
Cavernous sinus invasion	No	Reference	0.507	Reference	0.146	Reference	0.742
	Yes	2.43 [0.19 – 6.06]		0.28 [0.04 - 1.50]		0.68 [0.05 – 6.59]	
Suprasellar invasion	No	Reference	0.022*	Reference	0.994	Reference	0.018*
	Yes	48.3 [2.81 - 303]		12.3 [0.77 - 25.4]		52.5 [3.31 - 365]	

## Discussion

In this study, we introduced a GAN-based image synthesis method for generating synthetic DWI associated images using conventional MR images as the reference for model training. The synthetic images not only closely resembled real images in appearance but also demonstrated potential clinical value. Specifically, the synthetic images were effective in predicting tumor consistency and showed correlations with surgical approaches, outcomes and complications. These findings suggest that the image-to-image translation technique may offer additional diagnostic and prognostic utility beyond conventional imaging.

Previous studies have investigated the relationship between imaging features and the biological characteristics of pituitary adenomas. While enhanced T1-weighted imaging (T1WI) has shown that harder adenomas often exhibit more heterogeneous enhancement patterns, its diagnostic accuracy remains low [18]. DWI, which measures the Brownian motion of water molecules in tissues and quantifies it using the ADC, has demonstrated significant potential for preoperative assessment. Texture features derived from ADC images have been particularly effective in distinguishing soft from hard adenomas, with reported accuracy rates of 80–90% [6–9]. Additionally, Spearman analysis has revealed a significant correlation between ADC ratios and tumor collagen content, suggesting DWI’s utility in evaluating both tumor consistency and collagen composition [7,8].

Additionally, other imaging modalities, such as contrast-enhanced FIESTA [19] and MR elastography [20,21], may provide even more detailed information about the tissue properties of pituitary adenomas compared to conventional MR sequences, potentially improving prognosis prediction and aiding in surgical planning. However, despite the clear clinical benefits of these advanced MR techniques, their routine implementation in initial patient evaluations remains limited, which could impact preoperative decision-making.

Modern technology, using synthetic imaging, has been explored in other diseases, demonstrating its potential to enhance clinical decision-making. For instance, GAN-based image-to-image translation techniques have been used to generate accurate synthetic cerebral blood volume maps from standard MR scans, significantly aiding in the clinical evaluation of brain tumors [11]. AI-based models have also been shown to generate synthetic methionine PET images that strongly correlate with real PET images, effectively supporting glioma grading and prognostication [12]. Similarly, deep learning-based MR reconstruction has led to shorter examination times and improved image quality, maintaining diagnostic confidence in shoulder imaging compared to standard MR images [13]. These synthetic images not only closely resemble real images but also provide valuable clinical insights.

Preoperative prediction of tumor consistency not only assists patients in choosing specialized centers for treatment but also enables surgeons to customize surgical approaches accordingly. The GTR rate was significantly higher in the soft tumor group compared to the fibrous group. Our findings in the external cohort using synthetic MR images were consistent with these observations, reinforcing its potential clinical utility. Collectively, these results suggest that synthesizing images from standard MR images could offer a practical and efficient tool for diagnostic decision-making. In future studies, synthetic MR images may hold promise for evaluating tumor metabolism or even tumor microenvironments using similar techniques.

This study has several limitations. First, real DWI data were not obtained from external centers, which restricted the ability to compare the similarity between synthetic images and real ones from those institutions. However, this limitation underscores the potential utility of our synthetic images, as they could serve as a diagnostic supplement in centers lacking access to advanced imaging techniques. Additionally, the synthetic images proved valuable in predicting tumor consistency across these external centers. Second, tumor consistency was treated as a homogeneous characteristic by averaging values across the tumor, while in reality, tumor consistency can vary within a single tumor. This assumption may have impacted the accuracy of the synthetic maps in predicting surgical outcomes. Third, while we observed a strong correlation between imaging features and tumor consistency, the underlying biological mechanisms were not thoroughly investigated. There are hypotheses suggesting that both conventional T2-weighted imaging and DWI scans reflect stromal content (as we illustrated in the Masson’s staining), which may explain why our AI model could generate synthetic images closely resembling real ones from standard MR scans. Finally, despite the presence of domain shift—evidenced by variations in the prevalence of fibrous tumor consistency across centers—the model demonstrated robust performance in predicting tumor consistency. Nevertheless, we acknowledge that potential vendor- or protocol-specific biases may still limit broader generalizability.

In conclusion, our study demonstrated the diagnostic accuracy and clinical value of the GAN-based image synthesis method. This technique enabled precise prediction of tumor consistency and has the potential to guide surgical decision-making. Further investigations are warranted to explore the broader applicability of this technique in translating other clinically useful modalities for patients with pituitary adenomas.

## Supporting information

S1 Method Supplement method. (DOCX)

S1 Fig The network architecture of the generator. (DOCX)

S2 Fig The network architecture of the attention residual block. (DOCX)

S3 Fig The selected features and their importance rankings. (DOCX)

S4 Fig Calibration curve for the combined model in the independent test set. (DOCX)

S5 Fig Calibration curve for the combined model in the external cohort. (DOCX)

S6 Fig Decision curves for the models in the independent test set. (DOCX)

S7 Fig Decision curves for the models in the external cohort. (DOCX)
