# Supplementary material for: Generative adversarial networks-based Image-to-Image translation allows tumor consistency prediction from standard MR images in pituitary adenomas
Source: PLOS Digit Health. 2026 May 13;5(5):e0001407. doi: 10.1371/journal.pdig.0001407 (PMC13170839; doi:10.1371/journal.pdig.0001407)
Supplement: S1 Method — (DOCX) [file pdig.0001407.s001.docx]

Supplement method

The network architecture is shown in Figure 1. It consists of a contracting path (on the left) and an expanding path (on the right). The contracting path includes the repeated application of $Conv 4\times4$layers, with each convolution followed by a rectified linear unit ($ReLU$) and a Batch Normalization Block. In each down-sampling step, the number of feature channels is doubled. Each step in the expansion path includes up-sampling of the feature maps, followed by a $4\times4$ transposed convolution that halves the number of feature channels, concatenated with the corresponding feature maps from the contracting path, with each convolution followed by a $ReLU$ and a Batch Normalization Block. Additionally, the contracting and expanding paths are connected by skip connections. The bottleneck layer of Unet is replaced by two ordinary $Conv 3\times3$blocks and three attention residual blocks.

The attention residual block helps the model to focus on both detailed features and enhance the global perspective. Such a design allows the network to pay attention to the tumor area in the input image as well as the global features. The attention residual network, based on the residual network, adds a self-attention branch that passes the feature map $x$(with$C, H ,W$) through three $Conv 1\times1$layers to obtain the feature maps $Q, K, V$. (The channel number of $Q$ and $K$ is halved, while $V$ remains unchanged.) The transposed transformed $Q$ and $K$ are multiplied to obtain the correlation matrix $\beta$ between different channels, and to prevent the inner product from becoming too large, it is divided by the square root of $d_{k}$. In addition, the correlation matrix is normalized using the Softmax function and then multiplied by$V$ to obtain a feature map with global connections.

$$\text{Attention }(Q,K,V)=\mathrm{softmax}(\frac{QK^{T}}{\sqrt{d_{k}}})V$$

To enhance multi-scale spatial expression capabilities, $x’$ is then passed through a $Conv 3\times3$ layer to obtain the output feature map $y$. Thus, the attention residual network can be summarized as follows:

$$y=x+Conv(Attention(Conv(x),Conv(x),Conv(x)))+Conv(Conv(x))$$

Our model includes two types of losses: adversarial loss [1] and cycle consistency loss, which are used to match the distribution of generated images with the data distribution in the target domain; and the cycle consistency loss prevents the learned mappings ${Syn}_{T\to S}$ and ${Syn}_{S\to T}$ from being contradictory.

For the mapping function ${Syn}_{S\to T}$：T→O, the discriminator ${Dis}_{T}$ attempts to minimize the following objective:

$$\begin{matrix} \mathcal{L}_{\text{GAN}}({Syn}_{S\to T},{Dis}_{T},S,T)=E_{t\sim p_{data}(T)} \left[ log{Dis}_{T}\left( t \right) \right]+E_{s\sim p_{data}(S)} [\log(1-{Dis}_{S}\left( {Syn}_{S\to T}(s) \right) \end{matrix}$$

This objective function is designed to encourage the discriminator ${Dis}_{T}$to correctly distinguish between real images from the target domain T and those that are synthesized from the source domain S by the generator ${Syn}_{S\to T}$ The first term encourages the discriminator to identify real images from the target domain, while the second term encourages it to reject the synthesized images that are generated by the mapping function ${Syn}_{S\to T}$ from the source domain.

The discriminator ${Dis}_{T}$ is designed to predict the label 1 for real images from the target domain and the label 0 for synthesized images from the target domain. At the same time, the synthesis network ${Syn}_{S\to T}$ tries to maximize this loss by synthesizing images that are indistinguishable from real target domain.

Similarly, the discriminator ${Dis}_{S}$ is designed to predict the label 1 for real images from the source domain and the label 0 for synthesized images from the source domain. Therefore, the source domain loss function, which aims to minimize ${Dis}_{S}$ and maximize ${Syn}_{T\to S}$, is defined as follows:

$$\mathcal{L}_{\text{GAN}}\begin{matrix} ({Syn}_{T\to S},{Dis}_{S},T,S)=E_{s\sim p_{data}(S)} \left[ log{Dis}_{S}\left( s \right) \right]+E_{t\sim p_{data}(T)} [\log(1-{Dis}_{S}\left( {Syn}_{T\to S}(t) \right) \end{matrix}$$

This loss function encourages the generator ${Syn}_{T\to S}$ to create images from the source domain that are so realistic that the discriminator ${Dis}_{T}$ cannot distinguish them from real images from the source domain, thus maximizing the probability that ${Dis}_{T}$assigns the label 1 to these synthesized images.

Relying solely on adversarial loss cannot guarantee that the learned function will map individual input s to the desired output t. To further narrow down the space of possible mapping functions, cycle consistency loss is used to encourage this behavior, with an additional loss term defined as the difference between the original image and the reconstructed image:

$$\begin{matrix} \mathcal{L}_{\text{cyc }}\left( {Syn}_{S\to T},{Syn}_{T\to S} \right) & =\mathbb{E}_{s\sim p_{\text{data }}\left( s \right)}\left[ \parallel{Syn}_{T\to S}\left( {Syn}_{S\to T}\left( s \right) \right)-s\parallel_{1} \right] \\ & +\mathbb{E}_{t\sim p_{\text{data }}\left( t \right)}\left[ \parallel{Syn}_{S\to T}\left( {Syn}_{T\to S}\left( t \right) \right)-t\parallel_{1} \right] \end{matrix}$$

Therefore, the total loss can be summarized as follows:

$$\begin{matrix} \mathcal{L}_{\text{cyc }}\left( {Syn}_{S\to T},{Syn}_{T\to S},{Dis}_{S},{Dis}_{T} \right) & =\mathcal{L}_{\text{GAN}}({Syn}_{S\to T},{Dis}_{T},S,T) \\ & +\mathcal{L}_{\text{GAN}}({Syn}_{T\to S},{Dis}_{S},T,S) \\ & +\mathcal{L}_{\text{cyc }}\left( {Syn}_{S\to T},{Syn}_{T\to S} \right) \end{matrix}$$

[1] I. Goodfellow, J. Pouget-Abadie, M. Mirza, B. Xu, D. Warde-Farley, S. Ozair, A. Courville, and Y. Bengio. Generative adversarial nets. In NIPS, 2014.
