# Supplementary figures and images for: Generative adversarial networks-based Image-to-Image translation allows tumor consistency prediction from standard MR images in pituitary adenomas
Source: PLOS Digit Health. 2026 May 13;5(5):e0001407. doi: 10.1371/journal.pdig.0001407 (PMC13170839; doi:10.1371/journal.pdig.0001407)

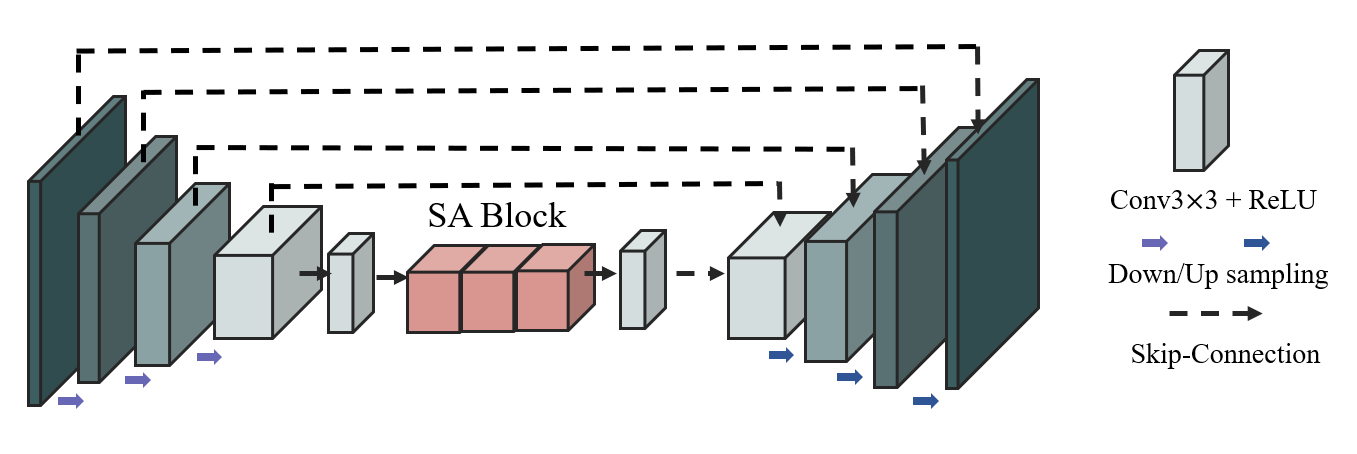


Fig. S1 The network architecture of the generator.

Supplement: S1 Fig — (DOCX) [file pdig.0001407.s002.docx]

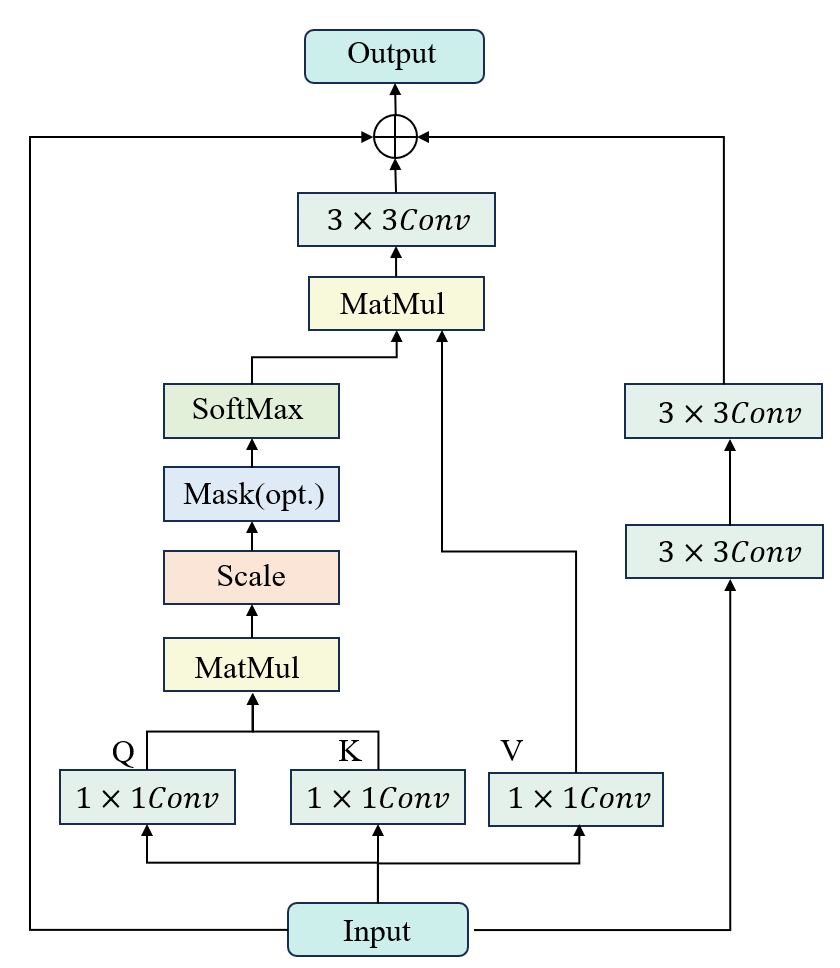


Fig. S2 The network architecture of the attention residual block.

Supplement: S2 Fig — (DOCX) [file pdig.0001407.s003.docx]

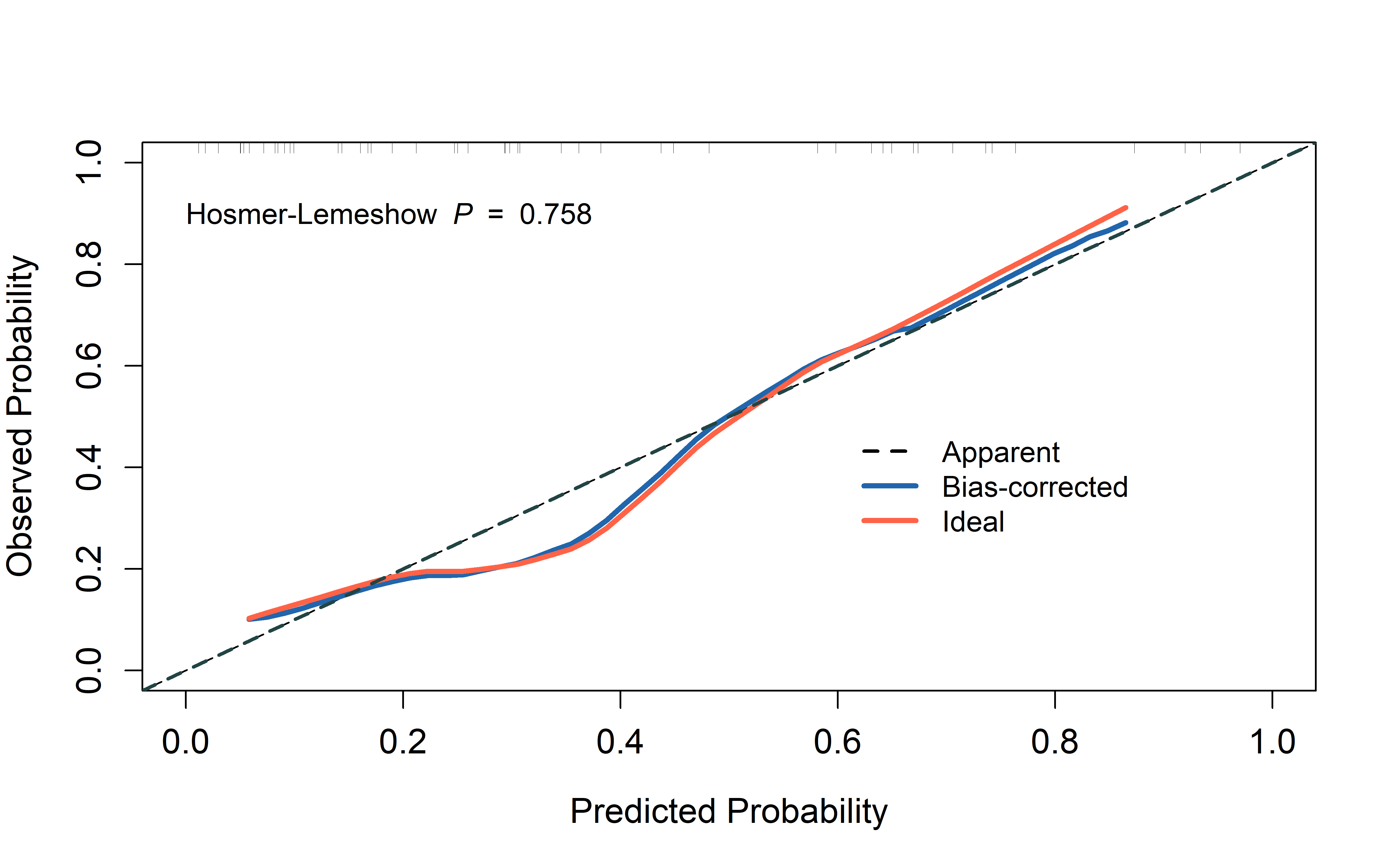


Figure S4. Calibration curve for the combined model in the independent test set

Supplement: S4 Fig — (DOCX) [file pdig.0001407.s005.docx]

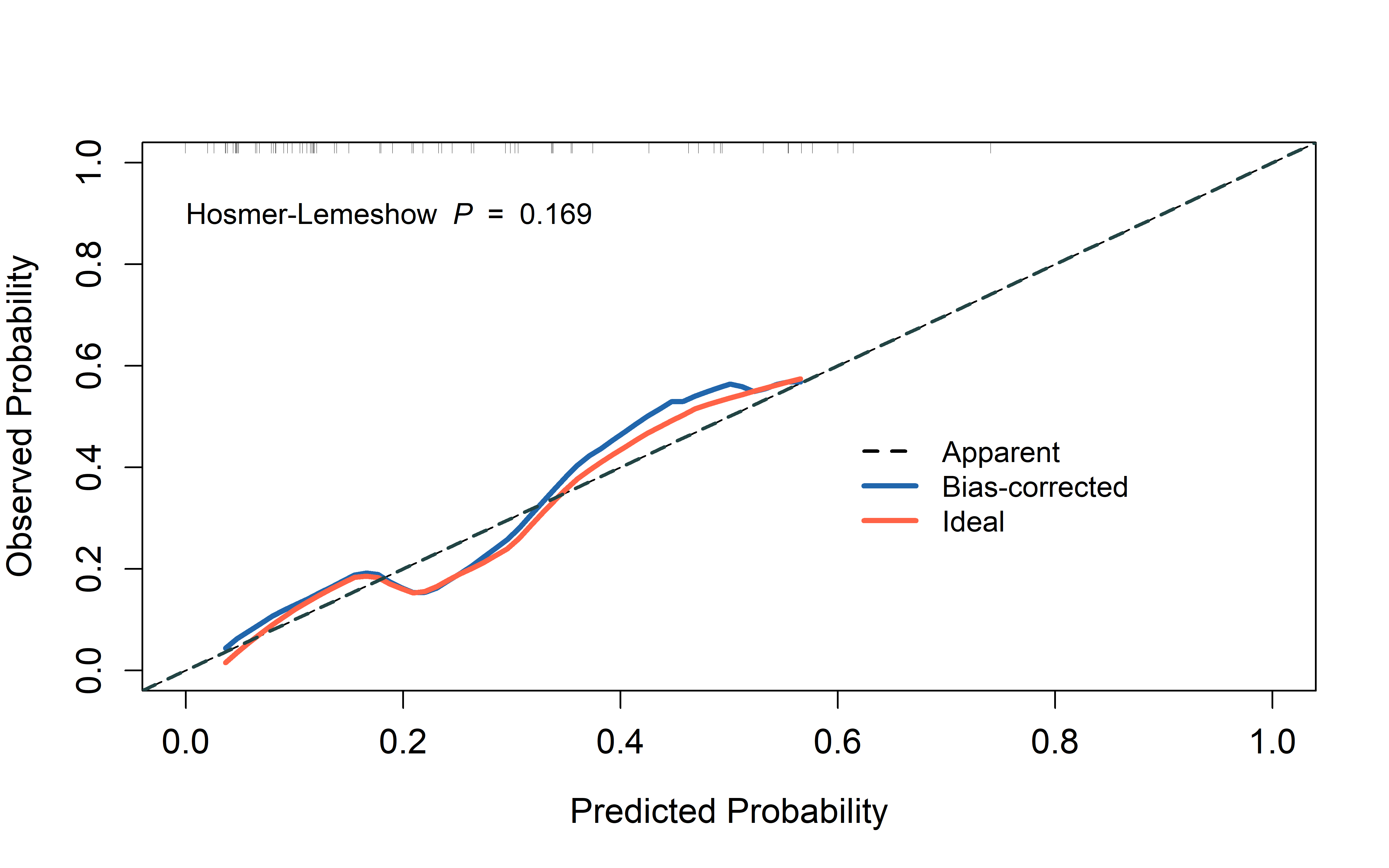


Figure S5. Calibration curve for the combined model in the external cohort

Supplement: S5 Fig — (DOCX) [file pdig.0001407.s006.docx]

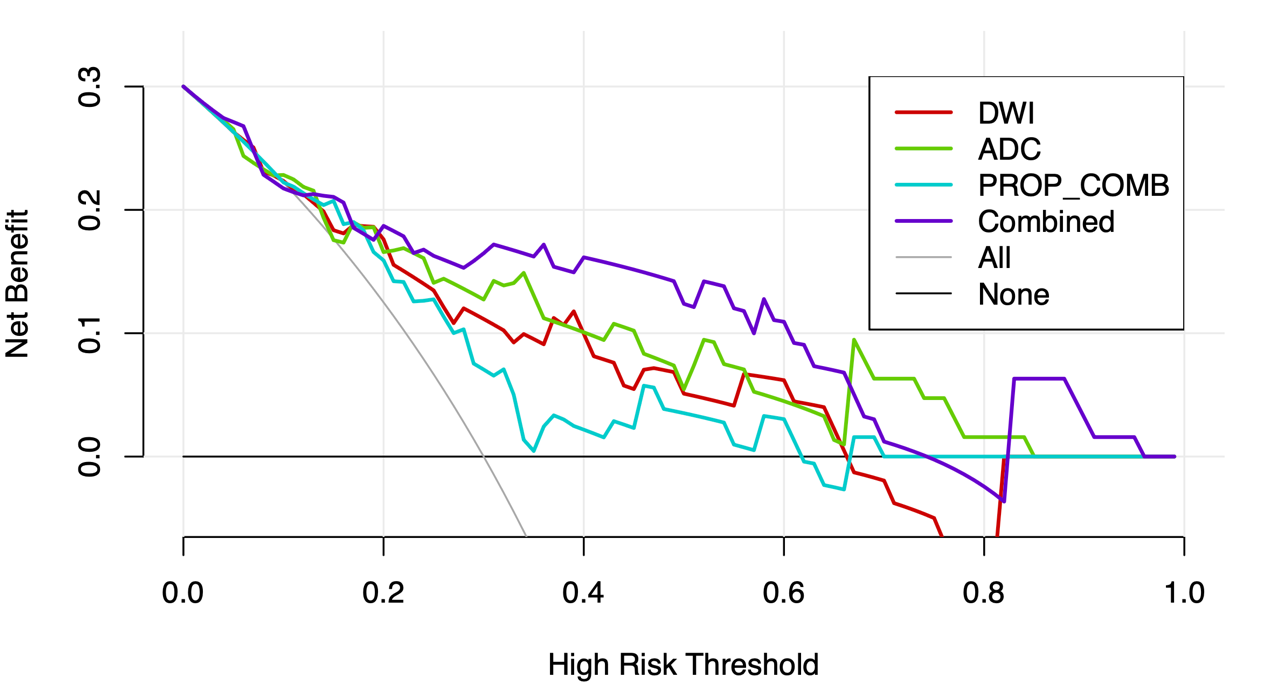


Figure S6. Decision curves for the models in the independent test set

Supplement: S6 Fig — (DOCX) [file pdig.0001407.s007.docx]

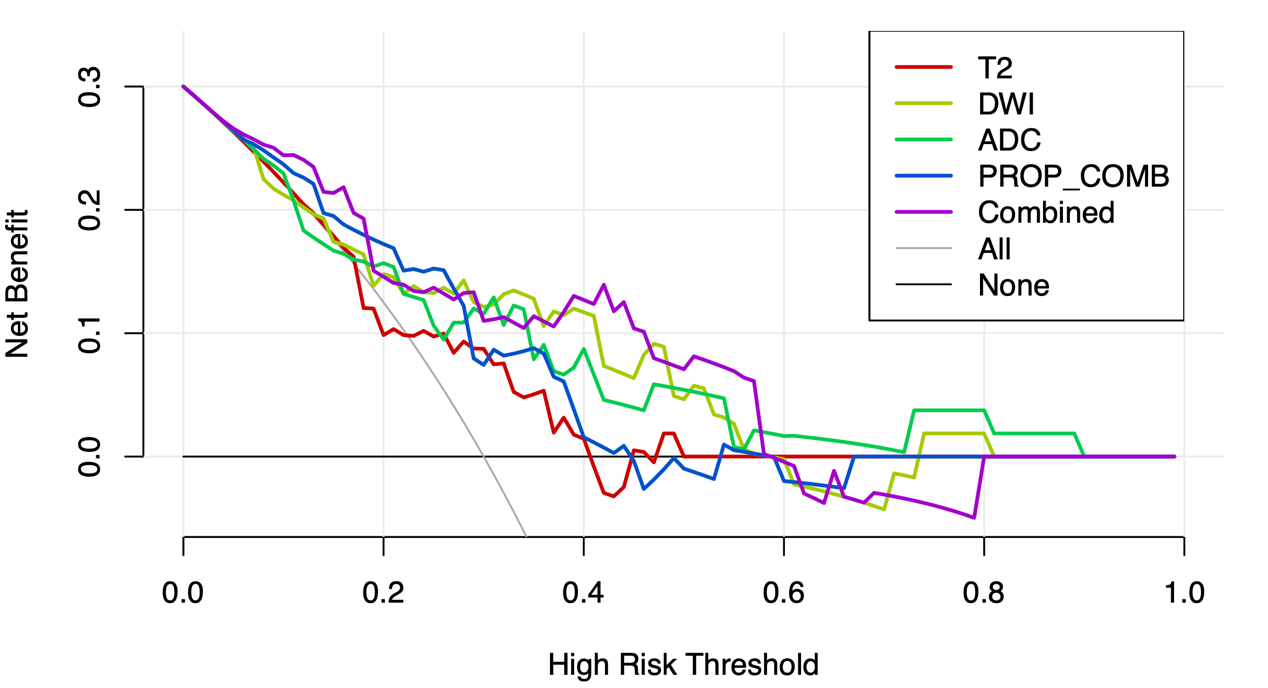


Figure S7. Decision curves for the models in the external cohort

Supplement: S7 Fig — (DOCX) [file pdig.0001407.s008.docx]
