# Supplementary material for: Generative adversarial networks-based Image-to-Image translation allows tumor consistency prediction from standard MR images in pituitary adenomas
Source: PLOS Digit Health. 2026 May 13;5(5):e0001407. doi: 10.1371/journal.pdig.0001407 (PMC13170839; doi:10.1371/journal.pdig.0001407)
Supplement: S3 Fig — (DOCX) [file pdig.0001407.s004.docx]

*
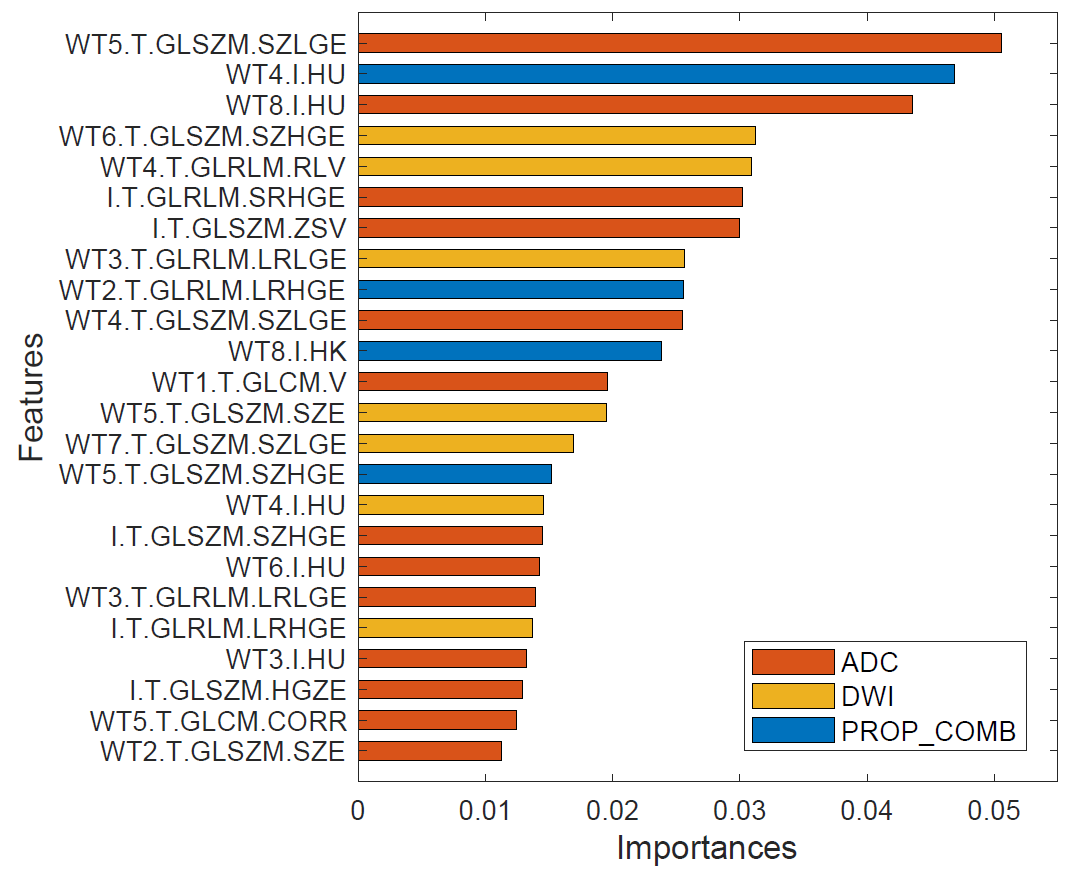
*

| **Abbreviation** | **Full name** |
| --- | --- |
| WT5.T.GLSZM.SZLGE | wavelet5.textures.gray-level size zone matrix.small zone low gray-level emphasis |
| WT4.I.HU | wavelet4.itensity.h-uniformity |
| WT8.I.HU | wavelet8.itensity.h-uniformity |
| WT6.T.GLSZM.SZHGE | wavelet6.textures.gray-level size zone matrix.small zone high gray-level emphasis |
| WT4.T.GLRLM.RLV | wavelet4.textures.gray-level run-length matrix.run-length variance |
| I.T.GLRLM.SRHGE | image.textures.gray-level run-length matrix.short run high gray-level emphasis |
| I.T.GLSZM.ZSV | image.textures.gray-level size zone matrix.zone-size variance |
| WT3.T.GLRLM.LRLGE | wavelet3.textures.gray-level run-length matrix.long run low gray-level emphasis |
| WT2.T.GLRLM.LRHGE | wavelet2.textures.gray-level run-length matrix.long run high gray-level emphasis |
| WT4.T.GLSZM.SZLGE | wavelet4.textures.gray-level size zone matrix.small zone low gray-level emphasis |
| WT8.I.HK | wavelet8.itensity.h-kurtosis |
| WT1.T.GLCM.V | wavelet1.textures.gray-level co-occurrence matrix.variance |
| WT5.T.GLSZM.SZE | wavelet5.textures.gray-level size zone matrix.small zone emphasis |
| WT7.T.GLSZM.SZLGE | wavelet7.textures.gray-level size zone matrix.small zone low gray-level emphasis |
| WT5.T.GLSZM.SZHGE | wavelet5.textures.gray-level size zone matrix.small zone high gray-level emphasis |
| WT4.I.HU | wavelet4.itensity.h-uniformity |
| I.T.GLSZM.SZHGE | image.textures.gray-level size zone matrix.small zone high gray-level emphasis |
| WT6.I.HU | wavelet6.itensity.h-uniformity |
| WT3.T.GLRLM.LRLGE | wavelet3.textures.gray-level run-length matrix.long run low gray-level emphasis |
| I.T.GLRLM.LRHGE | image.textures.gray-level run-length matrix.long run high gray-level emphasis |
| WT3.I.HU | wavelet3.itensity.h-uniformity |
| I.T.GLSZM.HGZE | image.textures.gray-level size zone matrix.high gray-level zone emphasis |
| WT5.T.GLCM.CORR | wavelet5.textures.gray-level co-occurrence matrix.correlation |
| WT2.T.GLSZM.SZE | wavelet2.textures.gray-level size zone matrix.small zone emphasis |

Figure S3. The selected features and their importance rankings.
